# miR-25-3p inhibition impairs tumorigenesis and invasion in gastric cancer cells *in vitro* and *in vivo*

**DOI:** 10.1080/21655979.2019.1710924

**Published:** 2020-01-07

**Authors:** Liang Ning, Maoshen Zhang, Qingli Zhu, Fengyun Hao, Wenlong Shen, Dong Chen

**Affiliations:** a Department of General Surgery, The Affiliated Hospital of Qingdao University, Qingdao, Shandong, China; b Department of Thyroid Surgery, The Affiliated Hospital of Qingdao University, Qingdao, Shandong, China; c Department of Pathology, The Affiliated Hospital of Qingdao University, Qingdao, Shandong, China; d Department of Anorectal, Qilu Hospital of Shandong University, Qingdao, Shandong, PR China

**Keywords:** Gastric cancer, miR-25-3p, miR-25 inhibitor, Metastasis, Invasion, microRNA

## Abstract

Deregulated expression of microRNAs (miRNAs) plays a role in the pathogenesis and progression of gastric cancer (GC). Among upregulated miRNAs, miR-25-3p has oncogenic potential and therefore represents an attractive target for the treatment of GC. Here, we investigated the role of miR-25-3p on GC cells *in vitro* and *in vivo*. We found that miR-25-3p overexpression significantly promoted growth and invasion of gastric cancer cells *in vitro*. Conversely, targeting miR-25-3p triggered significant inhibition of growth, invasion and migration in GC cells *in vitro. In vivo* delivery of miR-25-3p inhibitors induced significant anti-tumor activity in SCID mice bearing human GC xenografts. Our findings showed the evidence that *in vivo* antagonism of miR-25-3p impaired tumorigenesis, providing the rationale for clinical development of miR-25-3p inhibitors in GC.

## Introduction

Gastric carcinoma (GC) is the third leading causes of cancer-related deaths in the world [1]. It is known that high recurrence rate and rapid tumor growth have become the major disturbance to improve the survival of GC. Though, the treatment strategies of GC, such as surgical resection and chemotherapy, are effective way to increase therapeutic outcome, GC patients still have high risk and mortality [2]. Mounting evidence shows that many oncogenes or tumor suppressor gene are associated with GC development and progression.

miRNAs are a class of small, endogenous, non-coding RNAs that negatively regulate the expression of a wide variety of genes by binding to complementary sequences in the 3′-untranslated regions (UTRs) of target mRNAs [3,4]. A large number of studies have shown that miRNA alteration or dysfunction is involved in cancer development and progression by regulating cancer cell proliferation, differentiation, apoptosis, angiogenesis, metastasis, and metabolism [5]. Dysregulated miRNAs are involved in gastric cancer carcinogenesis and progression and function as oncogenes or tumor suppressors, as well as useful biomarkers in the diagnosis and prognosis of GC.

The miR-106b-25 cluster is highly conserved in vertebrates and consists of three members including miR-106b, miR-93 and miR-25. MiR-106b and miR-93 share the same seed sequences; however, miR-25 has only a similar seed sequence resulting in different predicted target mRNAs [6]. The mature miR-25 (miR-25-3p, accession numbe: MIMAT0000652) was used in the study. The mature miR-25 (miR-25-3p) consists of 22 nucleotides (CAUU GCAC UUGU CUCG GUCU GA) (www.miRbase.org) and has 1163 predicted target mRNA transcripts with conserved sites (TargetScanHuman version 7.1). Mature miR-25 belongs to the evolutionary broadly conserved miR- 25-3p/32-5p/92-3p/363-3p/367-3p seed family and has the same predicted mRNA targets as the other miRNA members of this seed family (TargetScanHuman version 7.1). MiR-25 is a well-described oncogenic miRNA playing a crucial role in the development of many tumor types including brain tumors, lung, breast, ovarian, prostate, thyroid, esophageal, colorectal, hepatocellular cancers [7].We have found that serum miR-25 level was significantly up-regulated in patients with GC, and high serum miR-25 level was significantly associated with depth of invasion, lymph node metastasis and stage of disease [8]. Li et al. showed overexpression of miR-25 in plasma and tissue samples of GC patients which promoted gastric cancer migration, invasion and proliferation by directly targeting the tumor suppressor TOB1 and correlated with poor survival [9]. Chen et al. has reported that miR-25 promoted TNBC cell proliferation *in vitro* and tumor growth in xenograft model, while suppression of miR-25 induced cell apoptosis [10]. However, as the isoform of mature miR-25, the biological role and underlying mechanisms of miR-25-3p in GC have not been well elucidated.

In this report, we first demonstrated the role of miR-25-3p overexpression on growth and invasion of GC cells *in vitro*. Then we demonstrated study the role of miR-25-3p inhibition on growth and invasion *in vitro*, and tumorigenesis and lung metastasis in GC cells *in vivo*, providing the framework for its clinical development.

## Materials and methods

### Cell culture

Human GC cell lines, BGC-823, MKN-28, SGC-7901, MGC-803 and one human gastric epithelial cell line GES-1were used in this study. All cell lines were obtained from Institute of Biochemistry and Cell Biology at the Chinese Academy of Sciences (Shanghai, China) and authenticated by the manufacturer. No additional authentication was performed by the authors for any of the cell lines. The cell lines were used between passages 2 and 10. The cell lines were grown in F-12 k (ATCC) supplemented with 10% fetal bovine serum and 1% penicillin-streptomycin at 37°C with humidified 5% CO_2._

### miR-25-3p overexpression or knockdown in vitro

Synthetic RNA molecules, including pre-miR-25-3p, miR-25-3p inhibitor (anti-miR-25-3p) and negative control RNA (miR-NC and anti-miR-NC) were purchased from GeneChem (Shanghai, China) and used for the overexpression and knockdown of miR-25-3p. miR-25-3p overexpression was achieved by transfecting cells with pre-miR-25-3p (a synthetic RNA oligonucleotide duplex mimicking miR-25-3p precursor), whereas miR-25-3p knockdown was achieved by transfecting cells with anti-miR-25-3p (a chemically modified single-stranded antisense oligonucleotide designed to specifically target against mature miR-25-3p). miR-NC and anti-miR-NC served as negative control. The sequences of synthetic pre-miR-25-3p was 5ʹ-CAUUGCACUUGUCUCGGUCUGA-3ʹ and 5ʹ-AGACCGAGACAAGUGCAAUGUU-3ʹ; The sequences of synthetic pre-miR-NC was 5ʹ-UUCUCCGAACGUGUCACGUTT-3ʹ and 5ʹ- ACGUGACACGUUCGGAGAATT -3ʹ; The sequences of synthetic anti-miR-25-3p was 5ʹ- UCAGACCGAGACAAGUGCAAUG -3ʹ; Anti-miR-NC was 5ʹ-CAGUACUUUUGUGUAGUACAA -3ʹ. After tsransfection for 24 h, cells were harvested and analyzed at the indicated times.

### Analysis of miR-25-3p expression using TaqMan reverse transcription PCR

Expression of miR-25-3p was analyzed using the TaqMan® MicroRNA Assays (Applied Biosystems). Expression of U6 (Applied Biosystems) was used as an endogenous control. miR-25-3p expression was measured relative to U6 (internal control) and quantified by the relative Ct method (2^ΔΔCt^). All the results are from 3 independent experiments done in duplicate. The TaqMan qPCR was carried out using LightCycler® 480 System (Roche) with the TaqMan universal PCR master mix (Applied Biosystems). All results from 3 independent experiments were performed in duplicate are presented as mean ± SEM (n = 3).

### In vitro *invasion and migration assays*

The invasion assay was performed with Matrigel (BD Biosciences, Sparks, MD, USA) coated on the upper surface of the transwell chamber (Corning, Lowell, MA, USA) [11]. The GC cells were seeded at 2.5 × 10^4^/well and transfected into pre-miR-25-3p, anti-miR-25-3p, miR-NC or anti-miR-NC for 24 h. Then, 500 μl complete endothelial cell growth medium (EGM-2 with growth factors) was placed in the lower wells serving as a source of chemoattractants. The cells were incubated for 36 h at 37°C. Cells migrated to the lower surface of the filter were fixed with 70% methanol and stained with 0.5% crystal violet solution. The number of migrated cells was determined by counting stained cells and the average cell number per field for each well was calculated. The counting was blinded by three individuals, including one who was blinded to the results. For each experiment, three to five replicate wells were used and the representative images were taken from five randomly selected fields of each well.

### In vitro *wound-closure assay*

A wound healing assay was utilized to evaluate tumor cell migration as previously described [12]. Briefly, the pre- miR-25-3p, anti-miR-25-3p, anti-miR-NC and miR-NC transfected GC cells was added to each well of a 6-well plate. When the cells reached close to 90% confluence, the cell monolayer was scraped in the center with a sterile plastic tip to generate a gap in the cell monolayer and then washed gently twice with media. Pictures of the plates were taken. At various time points, cell migration to the gaps in the center of each plate was monitored by taking photographic images of the plates, which were taken under an inverted microscope, and the data were analyzed at each time point based on sextuplet assays. Each experiment was repeated three times.

### Apoptosis assay

GC cells (5 × 10^5^ cells/ml) were transfected with pre-miR-25-3p or anti-miR-25-3p or anti-miR-NC for 72 h. Subsequently, cells were pelleted by centrifugation and incubated with Annexin V-FITC (Life Technologies, Shanghai, China) and 7-AAD (Becton Dickinson, Guangzhou, China). Cell suspensions were analyzed with an Attune Acoustic Focusing Flow Cytometer (Applied Biosystems). Cell death by apoptosis was scored by quantifying the population of Annexin V-FITC-positive cells using the FlowJo version 10 software.

### Cell proliferation assay

Proliferation of GC cells was determined by the colorimetric CellTiter 96 AQueous One Solution cell proliferation assay (Promega, Madison, WI, USA). Briefly, GC cells were cultured in RPMI 1640 supplemented with 10% fetal bovine serum (FBS), and 200 U/ml IL-2. The cells were harvested in their logarithmic phase (Cell passage 5 after thawing; Cell viability: ≥95%) and washed two times with the initial volume of Hanks’ balanced salt solution (HBSS) (by centrifugation at 1000 rpm, 5 min) and incubated for 4 h in assay medium (RPMI 1640 supplement with 10% FBS without IL-2) at 37°C, 5% CO_2_. During this period, a 96-well tissue culture plate was set up. A suspension of GC cells was adjusted to a concentration of 1 × 10^5^ cells/ml in media supplement with 10% FBS and added to the wells of a 96-well plate. Cells were grown overnight and transfected with pre-miR-25-3p and anti-miR-25-3p, anti-miR-NC and miR-NC. After the 24–72 h transfection period, CellTiter96® Aqueous One Solution was added (20 µl/well) and incubated for another 4 h at 37°C and 5% CO_2_ then 25 µl/well of 10% SDS was added. The plate was then read at 490nm. The background readings in the wells with medium were subtracted from the sample well read outs.

### Colony formation assay

pre- miR-25-3p, anti-miR-25-3p, anti-miR-NC and miR-NC transfected GC cells were plated at low density (2,500 cells per 10-cm plate), grown for 7–10 days. Then, the cells were washed with phosphate-buffered saline (PBS), fixed with 4% paraformaldehyde for 20 min and stained with a 0.5% crystal violet solution for another 20 min as described in ref [13]. The colonies were counted and imaged under a microscope. The experiments were replicated at least three times.

#### In vivo tumor formation and metastasis assays

Animal experiments were performed in compliance with the guidelines for the Welfare of Experimental Animals in the affiliated hospital of Qingdao University. For in vivo tumorigenicity assay, briefly, 1 × 10^7^ cells were subcutaneously into the right flank of each nude mouse, of 4- to 5-week old female BALB/c mice (Institute of Zoology, Chinese Academy of Sciences) (5 mice per group). Tumor volume was measured every 3 days over a 4-week period (formula: tumor volume (mm^3^) = length × width^2^ × 0.5). When the volume of xenograft tumor is approximately 100 mm3, the tumor was injected with naked anti-miR-NC or anti-miR-25-3p (30 ng, local injection) in combination with Invivofectamine 2.0 Reagent (Life Technologies) every 3 days following the manufacturer’s. After 3 weeks, the mice were sacrificed and the xenograft tumors were removed for formalin fixation and preparation of paraffin-embedded sections.

For in vivo metastasis assay, briefly, 1 × 10^6^ cells transfected with anti-miR-25-3p or anti-miR-NC were intravenously injected through the tail vein of 4- to 5-week-old nude mice (5 mice per group). After 4 weeks, the mice were euthanized and the number of metastases per lung was determined under a dissecting microscope. The lungs were excised and embedded in paraffin. Then, hematoxylin and eosin (H&E) staining was performed to affirm the presence of tumors.

### Statistical analysis

The version13.0 SPSS for Windows (SPSS Inc, IL, USA) and SAS 9.1 (SAS Institute, Cary, NC) softwares were used for statistical analysis. Values were expressed as means ± standard deviation. All experiments were done in triplicates at least two independent times. Statistical analysis was performed using Student’s t-test. A post hoc Tukey method was used to enable multiple comparisons between groups. Values of p < 0.05 were considered to be statistically significant.

## Results

### miR-25 expression in GC cell lines

**Figure 1.**
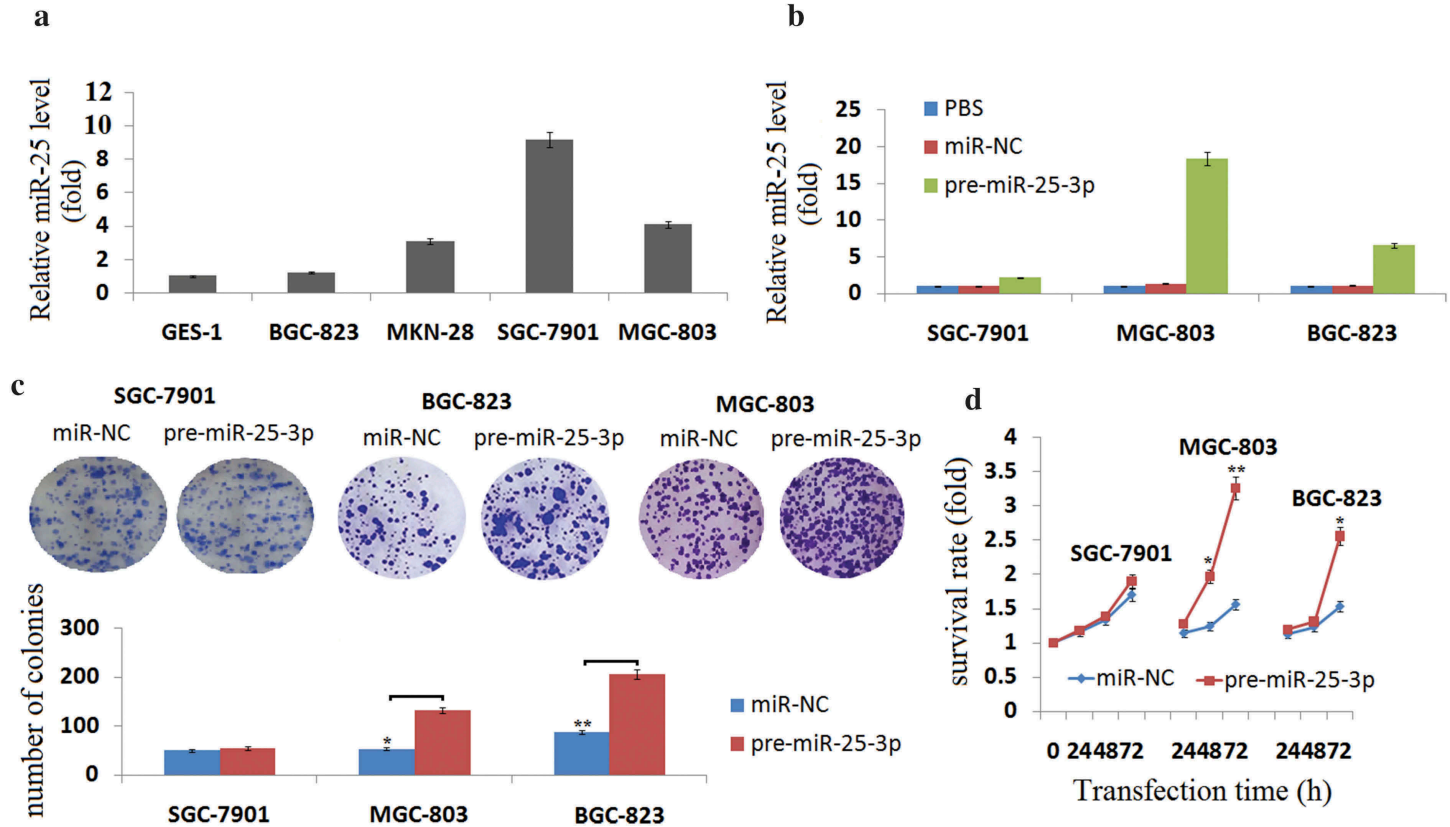
Enforced miR-25-3p promotes cell growth *in vitro.* (a) Quantitative RT-PCR analysis of miR-25-3p expression using total RNA from GC cells. Values represent mean ± SD of three different experiments. (b) Quantitative RT-PCR of miR-25-3p expression in SGC-7901, MGC-803, and BGC-823 cells cultured in the presence or absence of pre-miR-25-3p or miR-NC transfection. (c) Colony formation assay of SGC-7901, MGC-803, and BGC-823 cells transduced with a pre-miR-25-3p or miR-NC transfection for 10 days. Averaged values ±SD of three independent experiments are plotted. (d) Cell growth was measured by the colorimetric CellTiter 96 AQueous One Solution cell proliferation assay at 72 h. Viability was identical for cells treated with pre-miR-25-3p or miR-NC. The values are the means from three separate experiments done in triplicate. vs control, *P < 0.05; **P < 0.01.

By real-time PCR, we evaluated the miR-25 expression in BGC-823, MKN-28, SGC-7901, MGC-803 and human gastric epithelial cell line GES-1. Among these cell lines, we found variable miR-25 expression: SGC-7901 showed the highest, MGC-803 the modest and BGC-823 showed the lowest expression. (Figure 1(a)). In the present study, we used SGC-7901, MGC-803, and BGC-823 cells for further study.

### *Enforced miR-25 promotes cell growth* in vitro

SGC-7901, MGC-803, and BGC-823 cells were transfected into pre-miR-25-3p or miR-NC, respectively. Expression levels of miR-25-3p constructs were monitored and found to be 2,18 and 6 fold increase in SGC-7901, MGC-803 and BGC-823 fold increase above the endogenous levels in transduced cells respectively (Figure 1(b)). After transfection of pre-miR-25-3p, we observed that MGC-803 and BGC-823 cell lines formed larger colonies than controls when plated at low density (Figure 1(c)), and MGC-803 cells formed larger colonies compared to BGC-823 cells, however, SGC-7901 cell lines, which expressed the highest levels of miR-25, did not display significant changes (Figure 1(c)), suggesting that in some GC cells with high endogenous miR-25-3p, an increased miR-25-3p level does not stimulate growth.

To measure the impact of miR-25-3p on GC cell proliferation, a cell viability assay was used. As shown in Figure 1(d), treatment by pre-miR-25-3p for 24–72 h, but not miR-NC, increased cell growth in MGC-803, and BGC-823 cells. By contrast, no significant change in proliferation was observed in treated SGC-7901 cells.

### *Enforced miR-25 promotes cell invasion and migration* in vitro

**Figure 2.**
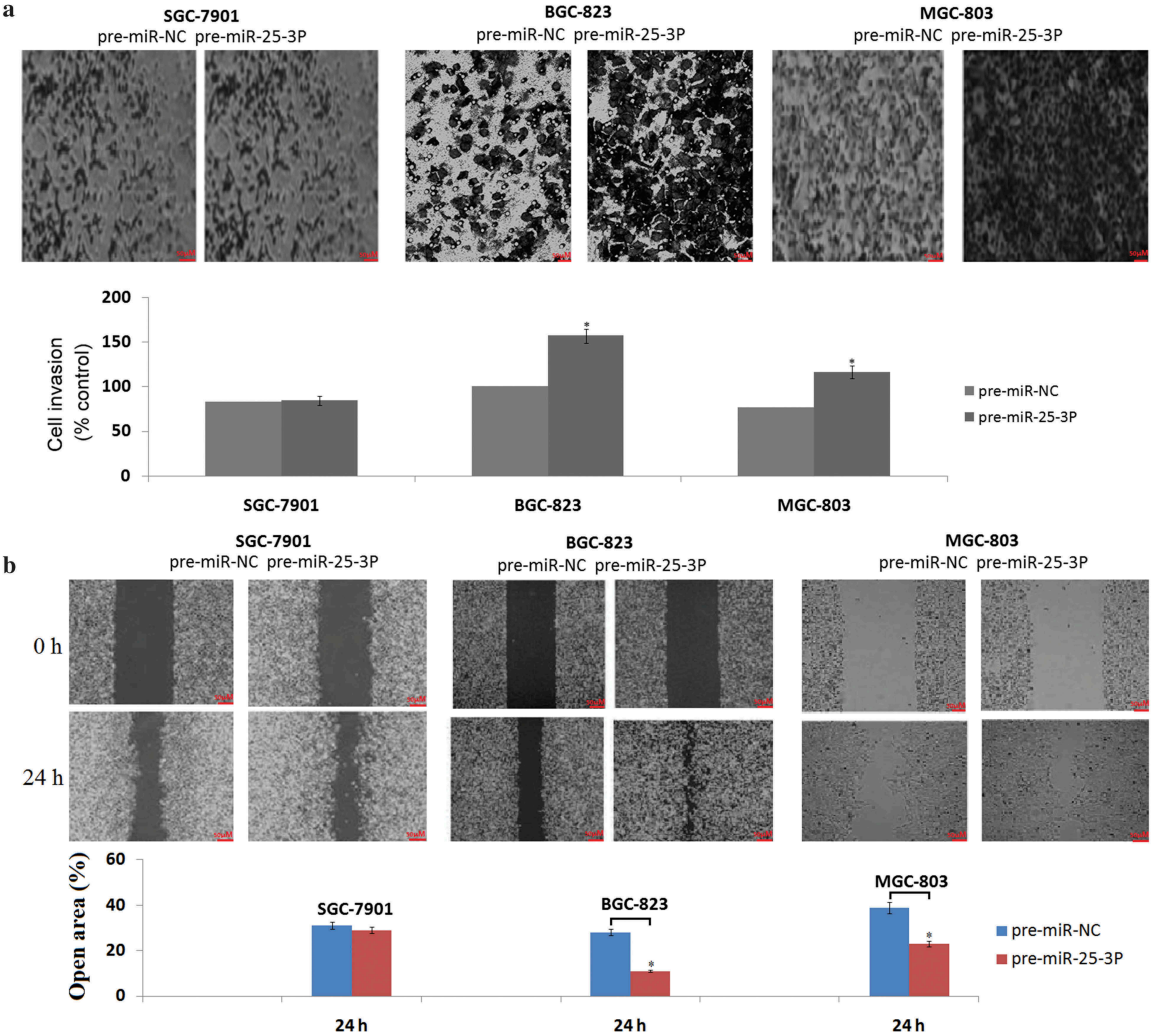
Enforced miR-25-3p promotes cell invasion and migration *in vivo.* (a)Anti-miR-25-3p transfected SGC-7901, MGC-803, and BGC-823 cells vs Anti-miR-NC transfected cells in a 200× light scope after crystal violet staining. (b) Cell migration was determined by the wound healing assay in SGC-7901, MGC-803, and BGC-823 cells with anti-miR-25-3p or anti-miR-NC transfection. Magnification: × 200. vs control, *P < 0.05;

We next determine the role of miR-25-3p overexpression on invasion and migration in SGC-7901, MGC-803, and BGC-823 cells through Transwell assays and Wound-healing assays. As shown in Figure 2(a-b), miR-25-3p dramatically enhanced the abilities of cellular invasion and migration in MGC-803 and BGC-823 cells. No significant effects of miR-25-3p overexpression on invasion and migration in SGC-7901 cells was observed (Figure 2(a-b)).

### *Targeting miR-25 inhibits cell growth* in vitro

**Figure 3.**
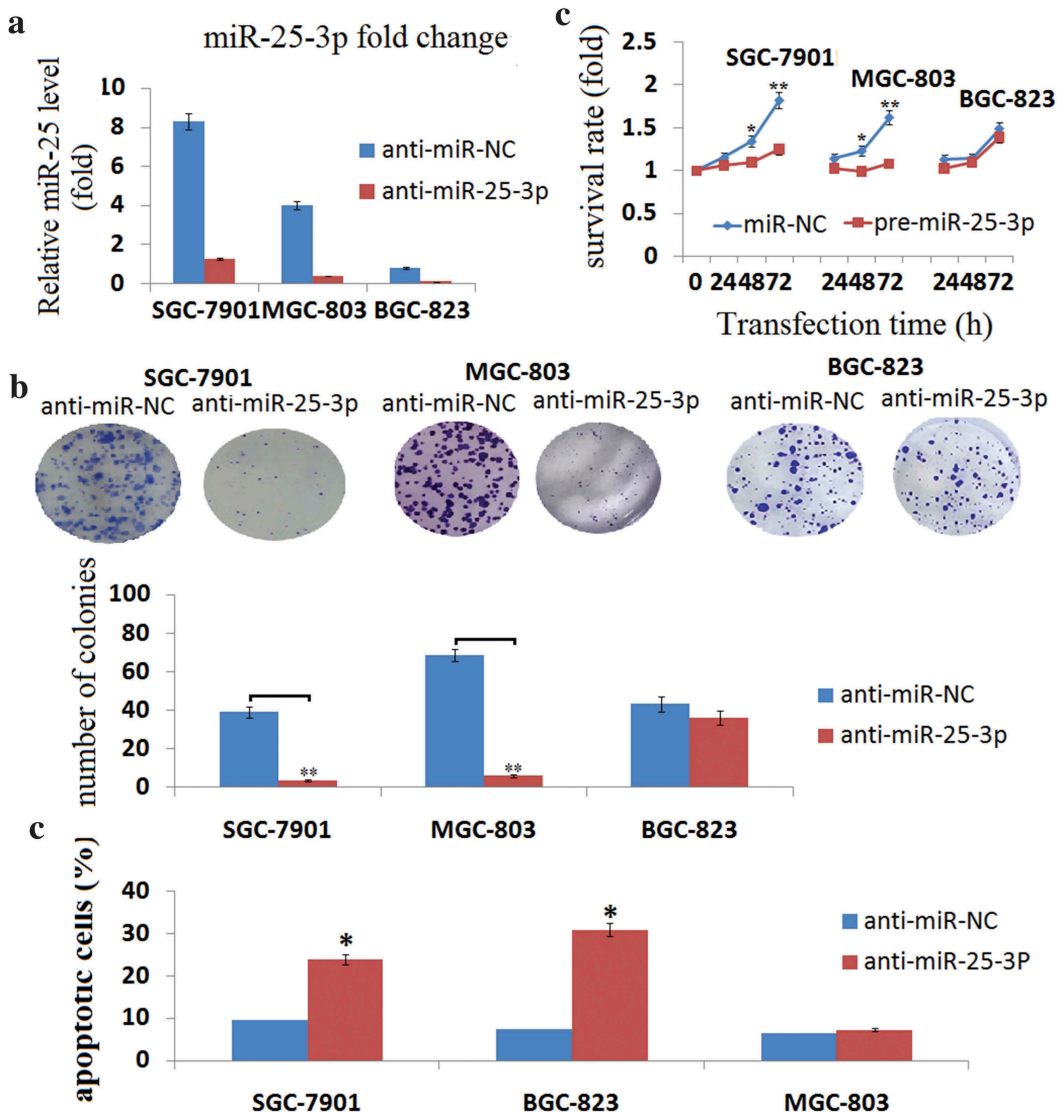
miR-25-3p inhibition impairs tumor cell growth. (a) Quantitative RT-PCR of miR-25-3p expression in SGC-7901, MGC-803, and BGC-823 cells cultured in the presence or absence of anti-miR-25-3p or anti-miR-NC transfection. (b) Cell growth was measured by the colorimetric CellTiter 96 AQueous One Solution cell proliferation assay at 72 h. Viability was identical for cells treated with anti-miR-25-3p or anti-miR-NC. The values are the means from three separate experiments done in triplicate. vs control, *P < 0.05; **P < 0.01.(c)SGC-7901, MGC-803, and BGC-823cells were plated at low density after transfection by anti-miR-25-3p or anti-miR-NC. Cells were grown for 10 days, fixed, and stained by crystal violet. vs control, *P < 0.05; **P < 0.01. (d)SGC-7901, MGC-803, and BGC-823 cells were cultured in the presence or absence of anti-miR-25-3p or anti-miR-NC for 72 h. Cell apoptosis was detected by Annexin V- FITC and 7-AAD dual staining . vs control, *P < 0.05; **P < 0.01.

To investigate the biological function of targeting miR-25 on growth, we performed loss- function experiments through transfection with anti-miR-25-25-3p and anti-miR-NC for 24 h in SGC-7901, MGC-803, and BGC-823 cells, respectively. As shown in Figure 3(a), a drastic reduction of miR-25-3p levels was observed by qPCR in high expressors of SGC-7901and MGC-803 cells, whereas a mild decrease was detected in BGC-823cell lines with low expression. As shown in Figure 3(b), treatment by anti-miR-25-25-3p, but not anti-miR-NC, reduced cell growth in SGC-7901 and MGC-803 cell lines overexpressing miR-25-3p. By contrast, no change in proliferation was observed in treated BGC-823 cells with less miR-25-25-3p expression.

We also observed that treatment by anti-miR-25-25-3p, but not anti-miR-NC formed smaller colonies in SGC-7901and MGC-803cells, however, BGC-823 cell lines, which expressed the lowest levels of miR-25-3p, did not display significant changes (Figure 3(b)), suggesting that in some GC cells with low endogenous miR-25-3p, an decreased miR-25-3p level does not inhibit growth.

Apoptosis is an important cause of tumor suppression. Flow cytometric analysis of apoptosis was performed to confirm the assumption that miR-25-3p functions as a candidate tumor promoter gene in GC. SGC-7901 and MGC-803 cells transfected with anti-miR-25-3p exhibited a higher rate of apoptosis compared with that of the anti-miR-NC control group (Figure 3(c)). Based on these findings, we suggested that targeting miR-25 accelerates the progression of GC cell apoptosis with high miR-25-3p expression.

### *Targeting miR-25 inhibits cell invasion and migration* in vitro

**Figure 4.**
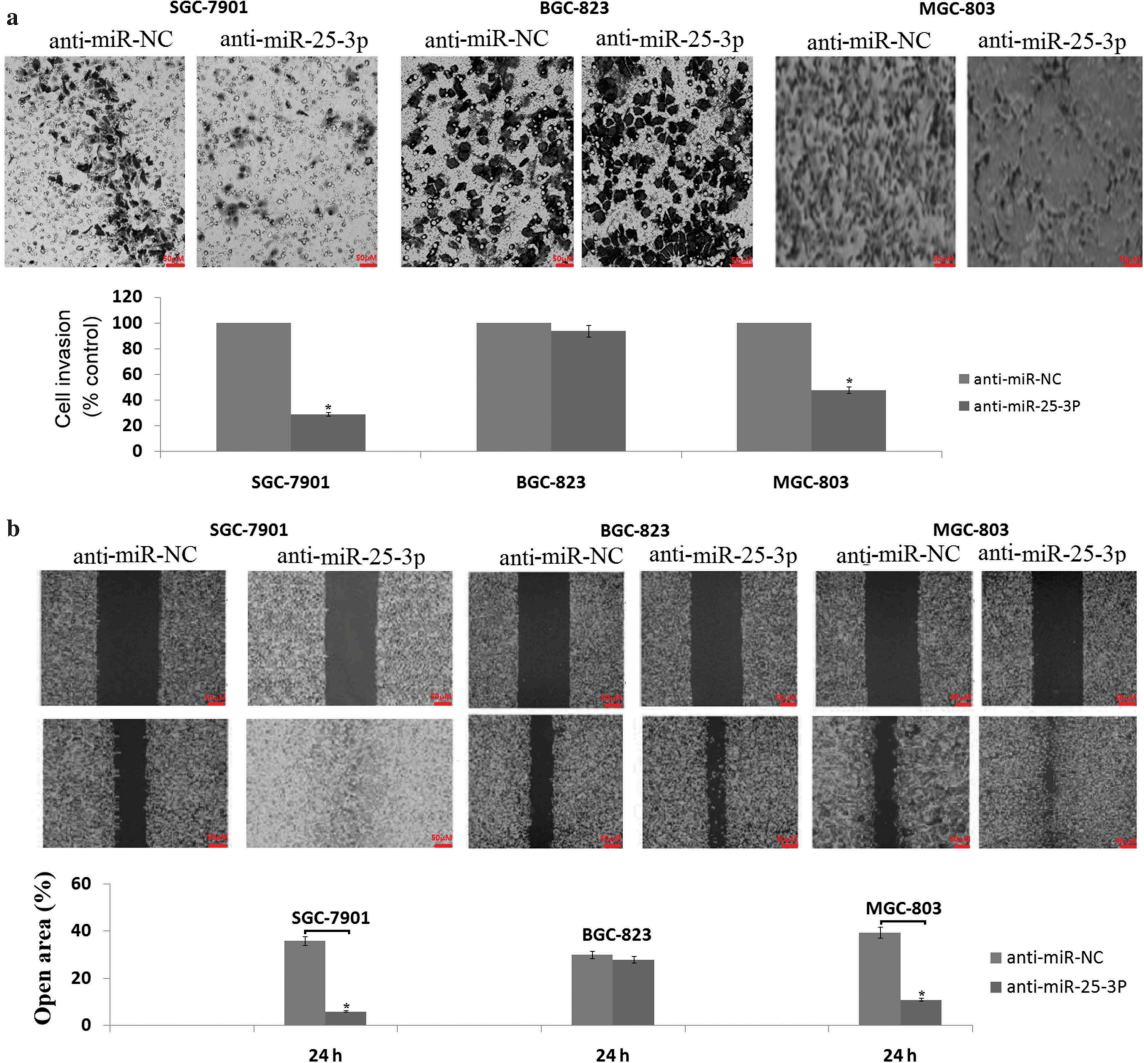
miR-25-3p inhibition impairs tumor cell invasion and migration. (a) anti-miR-25-3p or anti-miR-NC transfected SGC-7901, MGC-803, and BGC-823 cells in a 200× light scope after crystal violet staining. (b) Cell migration was determined by the wound healing assay in SGC-7901, MGC-803, and BGC-823 cells transfected with anti-miR-25-3p or anti-miR-NC. Magnification: × 100. vs control, *P < 0.05.

Transwell assays clearly revealed that targeting miR-25-3p significantly reduced the invasive activities of SGC-7901 and MGC-803 cells compared with the anti-miR-NC transfected SGC-7901 and MGC-803 cells (Figure4(a)). Wound-healing assays demonstrated that targeting miR-25-3p markedly weakened the migratory abilities of SGC-7901 and MGC-803 cells (Figure 4(b)). However, targeting miR-25-3p did not affect the invasive and migratory abilities of BGC-823 cells (Figure 4(a-b)). There is no significant difference of cell invasive and migrative ability in the untreated SGC-7901, BGC-823, MGC-803 cells, suggesting that the based miR-25-3p levels has no relation with GC cell invasion and migration.

### *Targeting miR-25-3p suppresses tumor growth and lung metastasis* in vivo

**Figure 5.**
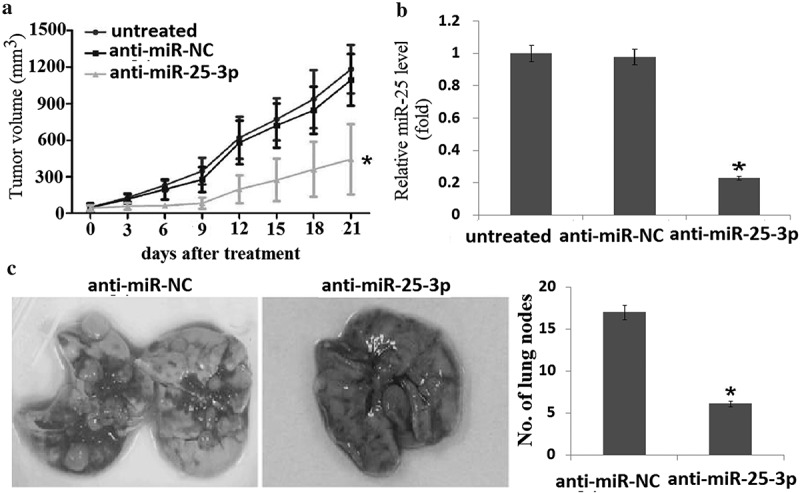
miR-25-3p inhibition impairs tumor formation and lung metastasis *in vivo.* (a)Growth curves of tumors obtained in SCID mice after subcutaneous inoculation of SGC-7901 cells, injected with PBS (25 μ L, local injection) or anti-miR-25-3p (30 ng, local injection) every 3 days for 21 days. (b) Quantitative RT-PCR of miR-25-3p expression in tumors in the presence or absence of anti-miR-25-3p. (c) Comparison of mean lung metastasis count for each group with SD. vs control, *,*P* < 0.05.

To investigate the effects of miR-25-3p on tumorigenicity *in vivo*, SGC-7901cells (1 × 10^7^) were injected into the flanks of nude mice to generate tumors ectopically. When the volume of xenograft tumor is approximately 100 mm3, the tumor was injected with anti-miR-NC or anti-miR-25-3p (30 ng, local injection) in combination with Invivofectamine 2.0 Reagent (Life Technologies) every 3 days for 3 weeks. As shown in Figure 5(a), the tumor volume in the anti-miR-25-3p group was decreased compared with those in the anti-miR-NC control groups or untreated groups. A drastic reduction of miR-25-3p levels was observed by qPCR in SGC-7901cells, whereas no decrease was detected in cell lines with anti-miR-NC transfection (Figure 5(b)).

To evaluate the in vivo effects of miR-25 on tumor metastasis, 1 × 10^6^ SGC-7901 cells transfected with anti-miR-25-3p or negative control were intravenously injected through the tail vein of 4- to 5-week-old nude mice (5 mice per group) for 4 weeks. Histological analysis revealed that the number of metastatic nodules was significantly reduced in the lung of mice injected with anti-miR-25-3p compared to that with of the mice injected with anti-miR-25-NC (Figure 5(c)). Taken together, these data indicated that targeting miR-25-3P inhibited growth and metastasis of gastric cancer cells overexpressing miR-25-3p *in vivo*.

## Discussion

In this report, we demonstrated that enforced miR-25-3p promoted growth and invasion in GC cells with relative low miR-25-3p expression *in vitro*. And antagonism of miR-25-3p by oligonucleotide inhibitors inhibited

tumorigenesis and metastasis *in vitro* and *in vivo* against human GC xenografts in SCID/NOD mice. To our knowledge, this is the first evidence of a successful *in vivo* treatment with miR-25-3p inhibitors in a murine xenograft model of human GC, which has important potential clinical applications. We show that efficacy of strategies based on miR-25-3p inhibition is dependent upon miR-25-3p expression levels in GC cells. Indeed, in GC cells expressing high miR-25-3p levels, miR-25-3p inhibitors reduce cell proliferation, survival, invasion and metastasis. In contrast, no significant effects were observed in cells with very low endogenous miR-25-3p expression. These data suggest that miR-25-3p expression is a potential biomarker predictive of therapeutic response to miR-25-3p inhibitors to be validated in future clinical trials. However, the broad of endogenous miR-25-3p expression where miR-25-3p inhibitors plays a effect role in needs further study.

The oncogenic role exerted by miR-25-3p in GC pathogenesis is predicted upon its upregulated levels even at early stages of disease. This notion is further supported by the role of miR-25-3p in FOXO3a and SLC34A2-miR-25-Gsk3β signaling, a central pathway for GC cell growth and drug resistance [14,15]. In this report, we demonstrate decreased cell proliferation and increased cell apoptosis in GC cells transfected with anti-miR-25-3p, and increased proliferation in GC cells transfected with pre-miR-25, further supporting this view. Others demonstrated that overexpression of miR-25-3p promoted cell proliferation, invasion and migration by directly down-regulating the tumor suppressor E3 ubiquitin ligase FBXW7 and up-regulating its substrates including G1/S-specific cyclin E1 (CCNE1) and v-myc avian myelocytomatosis viral oncogene homolog (MYC) in human GC samples and cell lines [16,17]. In our study, overexpression of miR-25-3p promoted invasion and migration, and targeting miR-25-3p inhibited invasion, migration and metastasis *in vitro* and *in vivo*, further supporting this view. Our data confirmed the tumor suppressive role of miR-25-3p through GC cells invasion, migration and apoptosis assays *in vitro*, along with tumor xenografts growth and metastasis *in vivo* according to both gain-of-function and loss-of-function experiments. However, the mechanisms that miR-25-3p functions need further investigation.

## Conclusion

In conclusion, our data offered the convincing evidences that miR-25-3p may function as a tumor promotor in human GC. miR-25-3p deregulation may inhibit proliferative, migration and invasion in GC cells. The newly identified miR-25-3p represents a novel potential therapeutic target for GC treatment.

## Research highlights

- Enforced miR-25-3p expression promotes growth and invasion of GC cells in vitro.
- Targeting miR-25-3p inhibits growth and invasion of GC cells in vitro.
- Targeting miR-25-3p inhibits tumorigenesis and lung metastasis of GC cells in vivo.
